# Enteric nervous system development: migration, differentiation, and disease

**DOI:** 10.1152/ajpgi.00452.2012

**Published:** 2013-05-02

**Authors:** Jonathan I. Lake, Robert O. Heuckeroth

**Affiliations:** ^1^Department of Pediatrics, Washington University School of Medicine, St. Louis, Missouri; and; ^2^Department of Developmental, Regenerative, and Stem Cell Biology, Washington University School of Medicine, St. Louis, Missouri

**Keywords:** enteric nervous system, development, neural crest, cell migration, chain migration, neurochemical coding, axonal targeting, neural crest-derived stem cells, Hirschsprung disease, pseudoobstruction, genetic interactions, gene-environment interactions

## Abstract

The enteric nervous system (ENS) provides the intrinsic innervation of the bowel and is the most neurochemically diverse branch of the peripheral nervous system, consisting of two layers of ganglia and fibers encircling the gastrointestinal tract. The ENS is vital for life and is capable of autonomous regulation of motility and secretion. Developmental studies in model organisms and genetic studies of the most common congenital disease of the ENS, Hirschsprung disease, have provided a detailed understanding of ENS development. The ENS originates in the neural crest, mostly from the vagal levels of the neuraxis, which invades, proliferates, and migrates within the intestinal wall until the entire bowel is colonized with enteric neural crest-derived cells (ENCDCs). After initial migration, the ENS develops further by responding to guidance factors and morphogens that pattern the bowel concentrically, differentiating into glia and neuronal subtypes and wiring together to form a functional nervous system. Molecules controlling this process, including glial cell line-derived neurotrophic factor and its receptor RET, endothelin (ET)-3 and its receptor endothelin receptor type B, and transcription factors such as SOX10 and PHOX2B, are required for ENS development in humans. Important areas of active investigation include mechanisms that guide ENCDC migration, the role and signals downstream of endothelin receptor type B, and control of differentiation, neurochemical coding, and axonal targeting. Recent work also focuses on disease treatment by exploring the natural role of ENS stem cells and investigating potential therapeutic uses. Disease prevention may also be possible by modifying the fetal microenvironment to reduce the penetrance of Hirschsprung disease-causing mutations.

the gastrointestinal tract requires finely tuned control over muscular activity and fluid secretion to efficiently break down macroscopic food particles, efficiently extract nutrients, and maintain a healthy luminal microbiome. An important arbiter of these processes is the enteric nervous system (ENS), a network of neurons and glia within the wall of the bowel that controls most aspects of intestinal function. In humans, the ENS contains ∼5 × 10^8^ neurons of >15 functional classes comprising a wide range of neurotransmitters, projection patterns, and electrical properties (71). When the ENS is missing (aganglionosis) or defective, children develop constipation, vomiting, abdominal pain, and growth failure and may die. Because ENS development and function are complex, the regulatory molecules that control ENS morphogenesis are also diverse. Disruption of one or more of these signals contributes to a spectrum of diseases. The ENS is derived from the neural crest (NC), a highly migratory and proliferative cell population originating at the junction of the neural plate and the adjacent ectoderm. NC cells invade the bowel and migrate through the mesenchyme in a process that is lengthy in distance traveled and time required. Failure of enteric NC-derived cells (ENCDCs) to colonize the distal bowel causes Hirschsprung disease (HSCR), a common (1 in 5,000 live births) and life-threatening developmental disorder. Because enteric neurons are required to actively relax intestinal smooth muscle, aganglionic bowel is tonically contracted, causing functional obstruction. HSCR is a non-Mendelian genetic disease with partial penetrance and variable expressivity. Several excellent reviews of HSCR genetics (2, 111) and ENS developmental biology (12, 40, 79, 81, 89, 90, 120, 140) have been published recently, but these fields are advancing rapidly. We review recent studies in the field in the context of existing models of ENS development and genetics and highlight areas that require additional investigation.

## Time Course of ENS Development

**Fig. 1.**
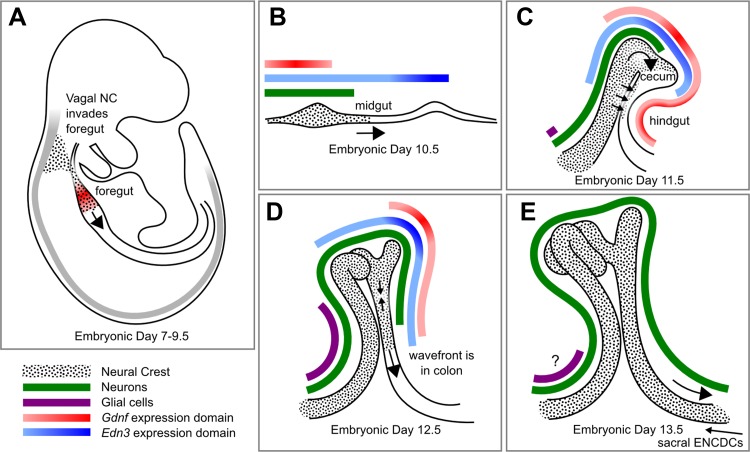
Initial colonization of the mouse gastrointestinal tract by enteric neural crest (NC)-derived cells (ENCDCs). *A*: during neural tube closure, NC cells (black) delaminate from the vagal region of the dorsal neural tube and migrate (arrows denote direction) in the ventral stream to the region adjacent to the foregut, which expresses glial cell line-derived neurotrophic factor (GDNF). *B–E*: after these pre-ENCDCs invade the foregut, they migrate rostrocaudally, proliferate, and differentiate first into neurons (green) and later into glia (purple: earliest glial marker brain fatty acid-binding protein). As this process proceeds, the bowel lengthens and changes shape, from a straight line (*B*) to a single bend with midgut and hindgut closely apposed (*C*); then the cecal appendage grows, and the entire bowel lengthens further (*D* and *E*). At embryonic *days 11* and *12*, ENCDCs invade the colon by crossing the mesentery and transiting the cecum (*C*). Cecal and transmesenteric populations then fuse to form the enteric nervous system (ENS) in the rostral colon (*D*), and the transmesenteric population populates the terminal colon as the smaller sacral ENCDC population enters the bowel and migrates caudorostrally (*E*). Regions of peak *Gdnf* (red) and endothelin 3 *(Edn3)* (blue) production are shown (*A–E*). Peaks of *Gdnf* expression partially, but imperfectly, mirror the extent of ENCDC migration, while peak *Edn3* expression is centered at the cecum. A smaller domain of *Gdnf* expression in the antimesenteric side of the terminal colon may attract ENCDCs across the mesentery (*C*). Human ENS development proceeds through a similar process.

**Fig. 2.**
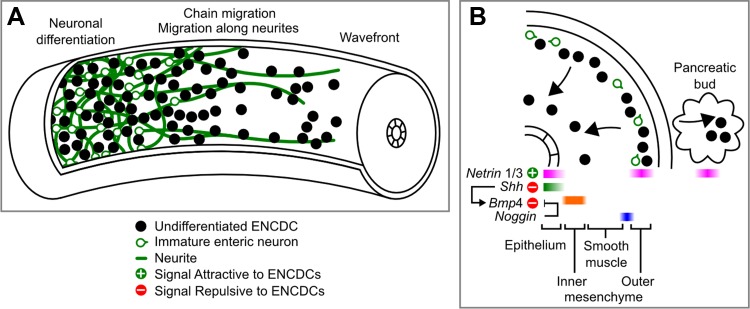
Primary and secondary migration of mouse ENCDCs. While the wave front of ENCDCs in the bowel moves steadily rostrocaudally, individual ENCDCs have complex and unpredictable behaviors. At and immediately behind the wave front (*A, middle* and *right*), ENCDCs migrate in chains and are often closely associated with the caudally projecting neurites of immature neurons, which extend up to the wave front. ENCDC connections are transient, and cells often swap neighbors within a chain or detach to switch chains or divide. Onset of neuronal lineage differentiation occurs very close to the wave front (*A, left*), and these cells retain some of their motility as they begin to extend neurites. In colonized regions in mice (*B*, cross-sectional illustration), a secondary centripetal migration of ENCDCs is triggered by trophic factors and the morphogens that control the patterning of the bowel wall. Netrin 1 and Netrin 3 are attractive to ENCDCs and are expressed in the epithelium, outer mesenchyme, and pancreatic buds, triggering the secondary migration of ENCDCs toward these structures. This broad attractive signal is probably refined by repulsive signals from sonic hedgehog (SHH) in the epithelium and later bone morphogenetic protein 4 (BMP4) expression in the inner mesenchyme, which SHH induces. A layer of BMP antagonist *Noggin-*expressing cells is located just inside the primary ENCDC migration layer, which may protect that region from the influence of BMP4. Precise timing of these signals in relation to each other and the secondary migration process have not been established. A similar secondary migration occurs in humans, but this process appears to proceed differently in birds.

ENS precursors originate in the vagal and sacral segments of the neural tube. The vagal NC is the major source of ENS precursors (217), while the sacral NC makes a small contribution to the distal bowel (28, 122a) and the anterior trunk NC makes a small contribution to the foregut ENS (57). Because vagal NC cells are the most extensively studied and form the vast majority of the ENS, we focus our discussion on the vagal NC while highlighting a few important differences in sacral ENCDC biology. At embryonic *day 9.5* in the mouse (108) and prior to *week 4* in human embryos (63), preenteric neural crest-derived cells (pre-ENCDCs) invade the foregut and begin their long rostrocaudal journey down the bowel. By embryonic *day 14* in mice and *week 7* in humans (66), this linear migration is complete (Fig. 1). In mice and humans, ENCDCs also undergo inward radial migration after initially colonizing the bowel (103), forming the two layers of ganglia that comprise the myenteric and submucosal plexuses (Fig. 2). Unless otherwise indicated, we refer to mouse gestational ages. As the ENCDCs migrate, they proliferate extensively and then differentiate into neurons and glia and condense into ganglia to form a network throughout the bowel. Recent data also suggest that ENS stem cells are present in fetal and adult mammals, raising interest in the possibility of autologous stem cell therapy for treatment of HSCR and other intestinal motility disorders (14, 138, 139). Formation of the ENS, therefore, requires extensive cell migration, controlled cell proliferation, regulated differentiation, directed neurite growth, and establishment of a network of interconnected neurons. Given these complex cellular events, each of which must be guided by specific molecular signals, it is not surprising that the genetics of ENS disease are complicated.

## Human Genetics of HSCR and Associated Clinical Syndromes

Most cases of HSCR are sporadic and occur as an isolated anomaly, but ∼20% are familial and 30% have cytogenetic abnormalities or additional developmental defects that constitute a recognizable clinical syndrome. At least 10 distinct genetic syndromes are strongly associated with HSCR, and many other disorders affecting genes without a clear role in ENS development occasionally include HSCR. These are beautifully discussed in recent reviews (2, 111). There is also a strong male predominance (4:1 male-to-female ratio) in children with HSCR restricted to the rectum and sigmoid colon (i.e., short-segment disease) and a weaker male predominance in children with long-segment disease. Sibling recurrence rates for HSCR vary from 1% to 33%, depending on the sex of the proband, the length of aganglionosis, and the sex of the new child. This is consistent with the hypothesis that affected females and those with longer aganglionic regions are more likely to carry greater genetic liability than males with short-segment HSCR. These complex genetic patterns are to be expected, given the developmental pathways needed to form the ENS and the many molecules that guide this development. Many mouse models with varying degrees of bowel aganglionosis, hypoganglionosis, and other defects have been isolated or engineered (Table 1); many of these are caused by disruption of orthologs of human HSCR genes.

**Table 1.** Genes affecting ENS development

**Genes Involved in RET and EDNRB Signaling**			
**Gene**	**Mouse model**	**ENS phenotype in mouse**	**Human disease association** (2)
*Ret* receptor tyrosine kinase	Monoisoformic alleles that are hypomorphic in the ENS despite not having any mutations:	Homozygous *Ret*^*miRet51/miRet51*^: colonic aganglionosis (48b) Hemizygous *Ret*^9/−^: colonic aganglionosis (199)	HSCR, total intestinal aganglionosis, MEN2A, MEN2B
	Serine phosphorylation site mutation *Ret*^*S697A*^	Homozygous: colonic aganglionosis (6)	
	Tyrosine phosphorylation site mutations such as	Homozygous: range of phenotypes from occasional hypoganglionosis to total intestinal aganglionosis. Effects of a given mutation depend on which isoform is mutated. Mutations affecting monoisomorphic RET9 have more deleterious effects than mutations affecting RET51	
	*Ret*^*Y1062F*^ (104)		
	*Ret*^*RET9(1062F)*^		
	*Ret*^*RET9(Y981F)*^		
	*Ret*^*RET9(Y1015F)*^		
	*Ret*^*RET51(Y1062F)*^		
	*Ret*^*RET51(Y1015F)*^ (102)		
	Missense MEN2A mutation *Ret*^*C620R*^	Homozygous: total intestinal aganglionosis Heterozygous: hypoganglionosis (35)	
	Dominant-negative allele: *Ret*^*RET9-L985P-Y1062F*^	Heterozygous: aganglionosis extending into the small bowel	
	Null alleles	Homozygous: total intestinal aganglionosis (175)	
		Heterozygous: subtle reductions in neuron size and fiber density. Abnormal bowel contractility (80)	
*Gdnf* neurotrophin, RET ligand	Null allele	Homozygous: total intestinal aganglionosis (172) Heterozygous: reduced enteric neuron density (80)	Mutations found in some HSCR cases
*Gfra1* RET coreceptor	Null allele	Homozygous: total intestinal aganglionosis (30)	
		Heterozygous: subtle reductions in neuron size and fiber density. Abnormal bowel contractility (80)	
*Nrtn* neurotrophin, RET ligand	Null allele	Homozygous: reduced soma size and fiber density in the myenteric plexus. Abnormal motility (94)	Mutations found in some HSCR cases
*Gfra2* RET coreceptor	Null allele	Homozygote: reduced fiber density and abnormal motility (169)	

*Ednrb* G protein-coupled receptor	Null allele: *Ednrb*^*s-l*^	Homozygote: colonic aganglionosis with hypoganglionosis of the small intestine (96)	HSCR, WS4
		Heterozygote: hypoganglionosis of the small intestine (33)	
	Hypomorphic allele: *Ednrb*^*s*^	Homozygote: rare colonic aganglionosis (136)	
*Edn3* EDNRB ligand	Null allele: *Edn3*^*ls*^	Homozygote: colonic aganglionosis (155)	WS4, very rare
*Ece1* EDN3 processing protease	Null allele	Homozygote: colonic aganglionosis (215)	1 case of HSCR with multiple birth defects
**Genes Involved in ENS Development and Implicated in Syndromic HSCR**			
*BBS1-11* intraciliary transport proteins	ENS not yet studied in mouse models. Morpholino knockdown in zebrafish causes ENS precursor migration defects (194)		Bardel-Biedl syndrome (±HSCR)
*KIAA1279 (Kbp)* unclear function	No mouse model exists. Zebrafish *kbp*^*st23*^ loss-of-function mutation reduces axon growth in the ENS (132)		Goldberg-Shprintzen syndrome (+HSCR)
*L1cam* L1 family cell adhesion molecule	Null allele	Transient ENCDC migration delay at *E11.5* (5)	X-linked congenital hydrocephalus, MASA syndrome (±HSCR)
*Pds5A* and *Pds5B* cohesin regulatory factor	Null alleles	Homozygotes: delayed ENS colonization (223), partially penetrant colonic aganglionosis (224)	Cornelia de Lange syndrome (1 family)
*Phox2b* homeodomain transcription factor	Null allele	Homozygous: total intestinal aganglionosis (154)	Congenital central hypoventilation syndrome, Haddad syndrome
*Sox10* SRY-related HMG-box transcription factor	Dominant-negative *Sox10*^*Dom*^	Heterozygous: colonic aganglionosis (117) Homozygous: total intestinal aganglionosis (109)	HSCR, WS4
	Null allele *Sox10*^*LacZ*^	Heterozygous: colonic aganglionosis (23)	
		Homozygous: total intestinal aganglionosis (23)	
*Zfhx1b* (SIP1, ZEB2) zinc-finger/homeo-domain protein	Null allele	Homozygous: failure of vagal NC delamination. ENCDCs do not enter the bowel (199b)	Mowat-Wilson syndrome (+HSCR)
**Genes Involved in ENS Development or Associated With HSCR**			
*Aldh1a2* (Raldh2) RA synthesis enzyme	Null allele	Homozygous: NC cells never enter the bowel (148)	
*Ascl1* (MASH1) basic helix-loop-helix transcription factor	Null allele	Serotonergic neurons absent from ENS (15), no neurons develop in the esophagus (85)	
*Dcc* receptor for netrin-1	Null allele	Homozygous: failure of ENCDCs to migrate to submucosal plexus and pancreas (103)	
*HOXB5* homeodomain transcription factor	Dominant-negative Tg(enb5), Tg(b3-IIIa-Cre), mosaic expression	Hypoganglionosis and aganglionosis of the ENS, *Ret* expression and migration reduced in the subset of cells that express dominant-negative *HOXB5* (131)	Variants associated with HSCR (37, 131)
*Ihh* hedgehog ligand	Null allele	Homozygous: ENS is absent in some regions of the small bowel and colon (165)	
*Kif26a-*negative regulator of RET signaling	Null allele	Homozygous: myenteric neuronal hyperplasia, pseudoobstruction (226)	
*Lgi4, Adam22* secreted factor and receptor involved in glial development and myelination	Null alleles	Homozygous: reduced numbers of glial cells, impaired glial marker expression, abnormal ENS structure (150)	
*NKX2-1* homeodomain transcription factor	ENS not studied in mouse models. Protein is detectable in human, but not mouse, ENCDCs		Mutations found in some HSCR cases (73)
*NRG1* ERBB3 ligand	ENS not yet studied in mouse models		HSCR (76)
*NRG3* ERBB4 ligand	ENS not yet studied in mouse models		HSCR (191)
*Ntrk3* (TrkC) receptor for NT-3	Null allele	Reduced numbers of enteric neurons, evidence for a selective reduction in late-born CGRP neurons (42)	Mutations found in some HSCR cases
*Ntf3* (NT-3) neurotrophin, TrkC/p75NTR ligand	Null allele	Reduced numbers of enteric neurons (42)	Mutations found in some HSCR cases
*Pax3* paired-box transcription factor	Null allele *Pax3*^*Sp*^	Homozygous: total intestinal aganglionosis (118)	Heterozygous mutations associated with WS without HSCR
*Phactr4* regulator of the actin cytoskeleton and cell adhesion	Mouse hypomorphic allele *Phactr4*^*humdy*^	Homozygous: colonic hypoganglionosis (225)	
*PROK1 PROKR1, PROKR2*	ENS not yet studied in mouse models. Receptors are expressed in cultured human enteric neurosphere-like bodies (171)		Mutations found in some HSCR cases
Prokineticin and receptors			
*Shh* hedgehog ligand	Null allele	Homozygous: ectopic neurons located in mucosa (165)	
*Slc6a2* (NET) norepinephrine reuptake transporter	Null allele	Homozygous: decreased neuronal numbers, selective decreases in numbers of serotonin- and calretinin-reactive neurons (125)	
*Tcof1* nucleolar factor	Null allele	Heterozygotes: delayed colonization of the bowel by ENCDCs. Migration continues between *E14* and *E18* to colonize the entire bowel	
*Tlx2* (Hox11L1) homeodomain transcription factor	Null allele	Homozygous: myenteric neuronal hyperplasia, pseudoobstruction (179)	
*Tph2* neuronal serotonin biosynthesis enzyme	Null allele	Homozygous: decreased numbers of myenteric neurons, selective decreases in numbers of dopaminergic and GABAergic neurons (126)	
*Spry2* regulator of receptor tyrosine kinases	Null allele	Homozygous: myenteric neuron hyperplasia, pseudoobstruction, achalasia (189)	
**Other Genes Associated With Syndromic HSCR**			
*DHCR7* final enzyme in cholesterol biosynthesis	ENS not yet studied in mouse models		Smith-Lemli-Opitz syndrome (±HSCR)
*RMRP* mitochondrial RNA-processing noncoding RNA	No viable mouse model		Cartilage-hair hypoplasia (±HSCR)
*TCF7L2*, (TCF4) transcription factor involved in Wnt signaling	ENS not yet studied in mouse models		Pitt-Hopkins syndrome (1 case), includes HSCR
**Transgenic Models Where Overexpression Alters ENS Development**			
**Mouse model**	**Description**	**ENS phenotype**	
Tg(DBH-NT3)	Ectopic neuronal and ENCDC expression of NT-3	Increased numbers of enteric neurons and neuronal hypertrophy (42)	
Tg(GFAP-GDNF)	Ectopic glial expression of GDNF	Increased numbers of submucosal neurons, increased numbers of nitrergic neurons, aberrant clustering of nitrergic axons around myenteric ganglia (208)	
Tg(HoxA4)	Global overexpression of homeodomain transcription factor	Colonic hypoganglionosis with neuronal hypertrophy (210)	
Tg(Mt1-GLI)	Ectopic and inducible expression of GLI1, activator of genes downstream of hedgehog pathway	Megacolon with hypoganglionosis, perinatal and adult death. Severity is related to expression level (216)	
Tg(NSE-Noggin)	Ectopic neuronal expression of BMP antagonist Noggin	Increased numbers of enteric neurons, with a selective decrease in the size of the TrkC-expressing population (38)	
**Conditional Mutations**			
**Gene**	Mouse model*	**ENS phenotype**	
*Cdh2* (*N*-cadherin) Homophilic cell adhesion molecule	Tg(Ht-PA-Cre) *Cdh2*^*LoxP*^	Delayed colonization of the colon. Severe migration defects in *Cdh2 Itgb1* double-conditional ENCDCs (24)	
*Dicer1* miRNA processing enzyme	Tg(Wnt1-Cre) *Dicer1*^*LoxP*^	Postcolonization loss of ENS cells (222)	
*Erbb2* EGF-receptor family member without known ligand heterodimerizes with ERBB3/4	Tg(Nestin-Cre) *Erbb2*^*LoxP*^ recombination in NC and other tissues, including colonic crypt epithelium	Postnatal loss of colonic neurons (46), thought to be due to loss of *Erbb2* in the epithelium, not the NC	
*Ercc1* nucleotide excision repair factor	Tg(Tyr-Cre) *Ercc1*^*LoxP*^	Postnatal death of colonic neurons (176)	
*Itgb1* (β_1_-integrin) cell-ECM adhesion molecule	Tg(Ht-PA-Cre) *Itgb1*^*LoxP*^	Colonic aganglionosis (22)	
*Hand2* basic helix-loop-helix transcription factor	Tg(Wnt1-Cre) *Hand2*^*LoxP*^	Tg(Wnt1-Cre), *Hand2*^*LoxP/LoxP*^ (93): disrupted patterning of nascent enteric ganglia and fiber network, reduction in neuronal density, failure of neurons to colocalize Tuj1 and Hu markers, selective loss of VIP-immunoreactive neurons	
		Tg(Wnt1-Cre), *Hand2*^*LoxP/null*^: loss of markers of terminal neuronal differentiation (Hu, microtubule-associated protein 2) and some neuronal subtypes (nNOS, dopamine β-hydroxylase). Fetal death at *E14* (48). More severe phenotype may be the result of heterozygosity for null allele (47)	
*Pofut1* required for Notch signaling	Tg(Wnt1-Cre) *Pofut1*^*LoxP*^	Hypoganglionosis (153)	
*Pten* phosphatase and tumor suppressor	Tg(Tyr-Cre) *Pten*^*LoxP*^	Hypertrophy and hyperplasia of enteric neurons (162)	
*Rac1* and *Cdc42* Rho family GTPases	Tg(Wnt1-Cre) *Rac1*^*LoxP*^ or Tg(Wnt1-Cre) *Cdc42*^*LoxP*^	Failure of ENCDCs to proliferate and colonize distal bowel (69)	
*Tfam* mitochondrial transcription factor	*CNP*^*Cre*^,*Tfam*^*LoxP*^ recombination in Schwann cells and ENS precursors	Postnatal death of specific subsets of enteric neurons (200)	
*Zfhx1b*(SIP1, ZEB2) zinc-finger/homeo-domain protein	Tg(Wnt1-Cre) *Zfhx1b*^*LoxP*^	Aganglionosis of the entire bowel distal to the stomach and rostral duodenum (199a)	
**Genetic Interactions in Model Systems**			
**Genes or alleles**		**ENS phenotype**	
*Ret*^+/−^	*Ednrb*^*s/s*^ *Ednrb*^*s-l/s*^	Highly penetrant aganglionosis in double-mutant animals (136). In isolation, *Ret*^+/−^ is not penetrant, and these Ednrb genotypes have extremely low penetrance	
*Ret*^*Y1062F/Y1062F*^	*Spry2*^*−*/−^	Partial rescue of nitrergic neuron density in the stomach. No effect on the remainder of the ENS (142)	
*Ednrb*^*sl/sl*^	*Ret*^*+/miRet51*^	Partial rescue: double-mutant embryos have a shorter aganglionic segment than *Ednrb*^*sl/sl*^ single mutants (8)	
*Sox10*^*Dom/+*^	*Ednrb*^*s/+*^ *Ednrb*^*s/s*^ *Ednrbl*^*s-l/+*^ *Ednrb*^*s-l/s-l*^	Double-mutant embryos have more penetrant aganglionosis (33), a more severe ENCDC developmental delay, and more pre-ENCDC cell death than *Sox10*^*Dom/+*^ embryos (183)	
*Sox10*^*Dom/+*^	*Edn3*^*ls/ls*^ *Edn3*^*ls/+*^	Double-mutant embryos have a more severe ENCDC developmental delay than *Sox10*^*Dom/+*^ embryos (183)	
*Sox10*^*LacZ/+*^	*Zfhx1b*^−/+^	Double-mutant embryos have a more severe ENCDC developmental delay and more extensive aganglionosis than *Sox10*^*LacZ/+*^ embryos (184). *Zfhx1b*^−/+^ genotype does not cause aganglionosis by itself	
*Sox10*^*LacZ/+*^	*Sox8*^*LacZ/+*^ *Sox8*^*LacZ/LacZ*^	Double-mutant embryos have a more severe ENCDC developmental delay, more extensive aganglionosis, and more pre-ENCDC cell death than *Sox10*^*LacZ/+*^ embryos (133). *Sox8* mutations do not affect ENS development in isolation	
*Sox10*^*LacZ/+*^	*L1cam*^+/−^/*L1cam*^*−/Y*^	Double-mutant embryos have a more severe ENCDC developmental delay, more extensive aganglionosis, and more pre-ENCDC cell death than *Sox10*^*LacZ/+*^ embryos (206). *L1cam* mutations individually produce transient delays in ENS development	
**Gene-Environment Interactions**			
**Genetic factor** *Ret*^+/−^ *Rbp4*^*−/−*^	**Environmental factor** Vitamin A deficiency during gestation	**ENS phenotype** Aganglionosis of the colon and small bowel. *Rbp4*^*−/−*^ mice depleted of vitamin A and *Ret*^+/−^/*Rbp4*^*−/−*^ mice fed vitamin A also developed less severe aganglionosis (65)	
*Tcof1*^+/−^	H_2_O_2_ exposure at *E7.5*	More severe ENCDC migration delay than *Tcof1* mutation alone. H_2_O_2_ had no effect on ENCDC migration in wild-type mice (9)	

Genes involved in Hirschsprung disease (HSCR) or known to be important to enteric nervous system (ENS) development are listed, and their mutant phenotypes are described. In addition, genetic interactions and gene-environment interactions that have been demonstrated in the mouse are listed. While many of the genes with well-documented roles in the ENS are also HSCR susceptibility genes, most are rare in humans. Conversely, the normal ENS developmental role of several HSCR susceptibility genes has not been explored. Human gene symbols are listed when mouse models have not been studied; otherwise mouse symbols are listed. While the *Hoxb5* dominant-negative mouse is a transgenic (Tg), it is listed together with the loss-of-function mutations because of the possible association of *HOXB5* with HSCR. Conditional mutations are listed here when they provide additional information about the role of each gene in ENS development. *Tg(Wnt1-Cre) and Tg(Ht-PA-Cre) lines result in recombination in the neural crest (NC), while the Tg(Tyr-Cre) line results in recombination in a subset of the vagal NC, including the ENS. Human chromosomal regions with as-yet-unidentified susceptibility loci and the genetic interactions that have been identified in humans are not included. BMP, bone morphogenetic protein; CGRP, calcitonin gene-related peptide; ECM, extracellular matrix; EDNRB, endothelin receptor type B; ENCDC, enteric NC-derived cell; *E7.5, E11.5, E14*, and *E18*, embryonic *days 7.5, 11.5, 14*, and *18*; Ihh, Indian hedgehog; MEN2A and MEN2B, multiple endocrine neoplasia 2A and 2B; nNOS, neuronal nitric oxide synthase; RA, retinoic acid; Shh, sonic hedgehog; WS, Waardenburg syndrome.

## Critical Molecular Mediators of ENS Development

The process of ENS development is controlled by cell surface receptors and their ligands, transcription factors that regulate their expression, morphogens, and proteins that transmit signals from the cell surface to the cytoskeleton and the nucleus. Very brief summaries of these proteins are provided before their role in cell biology and development is discussed.

### RET/GFRα1/GDNF pathway.

RET is a transmembrane tyrosine kinase receptor that is expressed in ENCDCs as they migrate through the bowel. It is the signaling receptor for four ligands [glial cell line-derived neurotrophic factor (GDNF), neurturin, artemin, and persephin] that activate RET by binding to the glycosylphosphatidylinositol-linked GDNF family of receptors (GFRα1, GFRα2, GFRα3, and GFRα4, respectively). RET signaling supports ENS precursor survival, proliferation, migration, differentiation, and neurite growth (80, 92, 95, 146, 192, 219). Heterozygous inactivating mutations in *RET* occur in ∼15% of children with sporadic HSCR and 50% of children with familial HSCR (2, 111). A common intronic enhancer polymorphism (RET +3, or rs2435357) is an important risk factor for HSCR that impairs *RET* expression (58). This polymorphism underlies many cases of HSCR because of its high prevalence in the population. In mice and humans, total RET deficiency causes complete intestinal aganglionosis, highlighting the central role of RET signaling in ENS development (175, 177). RET's coreceptor GFRα1 and ligand GDNF are the critical RET activators during fetal development, and loss of *Gdnf* and *Gfra1* causes phenotypes nearly identical to *Ret* in mutant mice. Indeed, these genes may be involved in rare cases of HSCR. Constitutively active mutations in *RET* cause the hereditary cancer syndromes multiple endocrine neoplasia type 2 (MEN2A and MEN2B) and familial medullary thyroid carcinoma. MEN2A is genetically heterogeneous and paradoxically associated with HSCR, despite mutations that constitutively activate *RET.* In contrast, MEN2B is almost always caused by the same M918T mutation and causes ganglioneuromas to form within the ENS, impairing bowel function.

### Endothelin receptor type B, endothelin-3, and endothelin-converting enzyme 1.

Another signaling pathway, centered on endothelin receptor type B (EDNRB) and its ligand endothelin 3 (ET-3), is required for ENS development in the colon. EDNRB is a G protein-coupled receptor expressed in NC derivatives, including the developing ENS. Hypomorphic or null mutations in *EDNRB, EDN3* (encoding the prepropeptide for ET-3), or the ligand-processing protease endothelin-converting enzyme (*ECE*) can cause HSCR, usually in the context of Waardenburg syndrome type 4 (WS4), a disorder that includes pigmentation defects, sensorineural deafness, dysmorphic facial features, and aganglionic megacolon in humans. Spontaneous mutation of EDNRB has also occurred in domesticated mice, rats, and horses, producing a similar phenotype.

### Transcription factors important for ENCDC colonization of the bowel.

Several transcription factors play critical roles in early ENS development. In part they are important because they influence the expression of *RET* (SOX10, PAX3, and PHOX2B) (118, 119, 124, 154) or *EDNRB* (SOX10) (228), but this is clearly not their only role. While these transcription factors are critical for cells in multiple organ systems, we concentrate on their roles in ENS development.

SOX10 is an SRY-related HMG-box transcription factor expressed in the neural tube prior to NC delamination, in migratory ENCDCs, and in mature enteric glia. In humans, heterozygous mutations in *SOX10* cause WS4 with a highly penetrant HSCR component (1, 10, 63). Experiments with homozygous *Sox10*-null mice revealed apoptotic cell death of NC cells prior to their entry into the foregut (109). Haploinsufficiency for *Sox10* appears to decrease the number of ENCDCs that initially colonize the bowel, eventually resulting in colonic aganglionosis. In addition to these requirements for survival, appropriate population size, and ENS gene transactivation, SOX10 has a critical role in maintaining ENCDCs in an undifferentiated state. Overexpression and loss-of-function experiments in primary cell culture (19, 112) and in chick embryos (137) indicate that SOX10 prevents precursors from differentiating into neurons.

PHOX2B is a homeodomain transcription factor expressed in the NC-derived autonomic nervous system, including the developing ENS and adult enteric neurons. PHOX2B is required for *Ret* expression in mouse pre-ENCDCs (154), and heterozygous *PHOX2B* polyalanine-expansion mutations cause congenital central hypoventilation syndrome (i.e., central sleep apnea) in humans, a syndrome that may include HSCR (Haddad syndrome) (2, 11).

Other transcription factors implicated in ENS development include PAX3 and ZFHX1B. In humans, heterozygous *PAX3* mutations cause WS4 without HSCR (158), but PAX3 is required for development of the ENS in mouse (118). PAX3 also activates *Ret* transcription in concert with SOX10. *ZFHX1B* (*ZEB2/SIP1*) mutations cause HSCR in the context of Mowat-Wilson syndrome, which also includes microcephaly, mental retardation, and dysmorphic facial features (31, 204). In mice, ablation of *Zfhx1b* within the NC prevents ENCDC migration beyond the proximal duodenum (199a). NKX2-1 and HOXB5 also physically associate with the promoter of *RET* and increase its expression (124, 227). Their necessity in vivo remains uncertain in the mouse, although mutations in both genes have been detected in the DNA of some HSCR patients (75, 131).

Several other transcription factors have been linked to the ENS in model systems but have unexpected mutant phenotypes or an unknown relevance to human disease. ASCL1 (MASH1) and HAND2 (dHAND) are transcription factors required for the development of subsets of autonomic neurons. In *Ascl1*^−/−^ mice, ENCDCs colonize the bowel but develop into a sparse and abnormal ganglionic network (15) and do not form serotonergic (15) or esophageal (85) neurons. Loss of *Hand2* (93) results in a complex phenotype involving a failure of multiple aspects of ENS development. *Ascl1* and *Hand2* are discussed in the context of neuronal subtype specification.

### Morphogens in ENS development.

Organization of the ENS requires the establishment of two ganglion cell networks in precise locations within the bowel wall (Fig. 2*B*). Neurons and glia cluster into ganglia, and then neurons extend neurites that initially fasciculate before innervating targets. Molecules controlling ENS morphogenesis are relatively poorly understood, but several classic morphogens are known to have important roles in ENS development. Some specific trophic factors are also critical for subsets of enteric neurons, but their absence does not cause intestinal aganglionosis or malformed ganglia.

The hedgehog pathway is involved indirectly and directly in the developing ENS. Hedgehog proteins have important roles as morphogens. For example, localized sonic hedgehog (SHH) expression is critical for defining anterior-posterior patterning of digits in the limb (10) and dorsoventral patterning in the spinal cord (50). Similarly, in the bowel, localized expression of hedgehog proteins in the epithelium is essential for concentric patterning of the bowel wall (165). The hedgehog ligands SHH and Indian hedgehog (IHH) are expressed by the gut epithelium during bowel development (13, 165) (Fig. 2*B*). However, loss of SHH or IHH has very different effects in mice, despite signaling through the same receptor and transduction machinery. Targeted mutation of *Shh* results in excessive numbers of enteric neurons and improper colonization of villi by enteric neuron cell bodies, whereas loss of *Ihh* causes dilated segments of bowel and aganglionosis in parts of the gastrointestinal tract (165). Oddly, ectopic expression of the hedgehog pathway's transcriptional effector GLI in developing mice produced an effect similar to loss of *Ihh* (216). These disparate phenotypes in mice with hedgehog signaling pathway mutations are incompletely understood. It is possible that *Ihh* and *Shh* mutant phenotypes differ because of the important temporal or spatial expression requirement for these proteins. Some of these phenotypes are consistent with known hedgehog effects on ENCDCs, since SHH promotes proliferation, inhibits neuronal differentiation, and prevents premature centripetal invasion of ENCDCs into the future submucosa (64, 187).

A second role for hedgehog in the developing ENS is indirect. Hedgehog signaling induces bowel mesenchyme to secrete bone morphogenetic protein 4 (BMP4), another important modulator of ENS patterning. During initial ENCDC migration, BMP4 expression is induced in a ring of mesenchyme adjacent to the epithelium. Noggin, a BMP antagonist, is secreted by cells surrounding the BMP4-producing mesenchyme (82) and presumably reduces the effect of BMP4 on migratory ENCDCs (67). Interestingly, BMP effects on ENCDC migration differ between mouse and chick. In organotypic and explant cultures of embryonic mouse bowel, inhibition of BMP4 signaling with Noggin enhances ENCDC migration (67), while chick embryos that overexpress Noggin in the mesenchyme inhibit ENCDC migration (82). However, BMP4 clearly enhances neuronal aggregation in both organisms (38, 64, 82) and is probably important for the clustering of ENCDCs into definitive ganglia. BMP4 also induces the fasciculation of neurites in cell and organotypic culture systems. BMP effects on aggregation and fasciculation appear to be mediated through the addition of the polysaccharide polysialic acid to neural cell adhesion molecule (NCAM) expressed by ENCDCs and enteric neurons (62, 67).

Netrins, diffusible ligands involved in central nervous system (CNS) and peripheral nervous system (PNS) patterning, are also involved in the radial migration of ENCDCs that occurs after initial colonization of the bowel. In mice, Netrin 1 and Netrin 3 are produced by the outer bowel mesenchyme in the presumptive myenteric region and by the intestinal mucosa and pancreatic buds (103), which are also invaded by ENCDCs during this secondary migration (Fig. 2*B*). Deleted in colon cancer (DCC), a netrin receptor, is expressed in migrating ENCDCs and is required for netrins to attract ENCDCs, since *Dcc*^−/−^ mice do not develop a submucosal plexus. Enteric neurons also produce netrins after they differentiate (167), attracting extrinsic fibers from the vagus nerve. Interestingly, laminin, an extracellular matrix (ECM) molecule that accumulates in the epithelial basal lamina and around enteric ganglia, converts the attractive effect of netrins on vagal axons to repulsion (166). It is unclear if this repulsion of fibers also applies to migrating ENCDCs, but if it does, such an effect could contribute to the cohesion of ganglia and the exclusion of ENCDCs from the epithelium.

Semaphorins are diffusible ligands involved primarily in axon growth cone repulsion. In the developing colon and cecum, semaphorin 3A (Sema3A) is expressed by the inner mesenchyme, while the coreceptor for Sema3A, neuropilin-1, is expressed in all ENCDCs (4). Despite its wide expression, Sema3A appears to specifically affect sacral ENCDCs and the extrinsic axons on which they migrate. Normally, sacral ENCDCs are sequestered within the pelvic ganglia that flank the end of the colon until embryonic *day 13.5*. They begin migrating up the colon, closely associated with extrinsic nerve fibers, just before arrival of the vagal ENCDC wave front (110, 209). In *Sema3A*^−/−^ embryos, sacral ENCDCs migrate into the colon early, demonstrating that Sema3A serves as a repulsive cue (4).

Retinoic acid (RA) is a diffusible morphogen produced locally in tissues by the retinaldehyde dehydrogenase (RALDH) enzymes. Mice lacking *Raldh2* die prior to ENS development, but viability can be prolonged by exogenous RA supplementation. These partially rescued embryos lack ENCDCs entirely (148), indicating a clear role for RA in ENS development. In vitro, RA has dramatic effects on ENCDCs and differentiating enteric neurons. For example, RA is required for the efficient migration of ENCDCs and acts by reducing levels of phosphatase and tensin homolog (PTEN) protein, a critical negative regulator of ENCDC migration and proliferation (see below). RA also induces shorter neurites in enteric neurons, a response opposite to that of most other neurons (174). Because RA is essential for normal ENS development, mouse embryos with impaired RA production due to deficiency in its dietary source, vitamin A, also have defects in ENS development (65).

### Intracellular signaling molecules in the developing ENS.

**Fig. 3.**
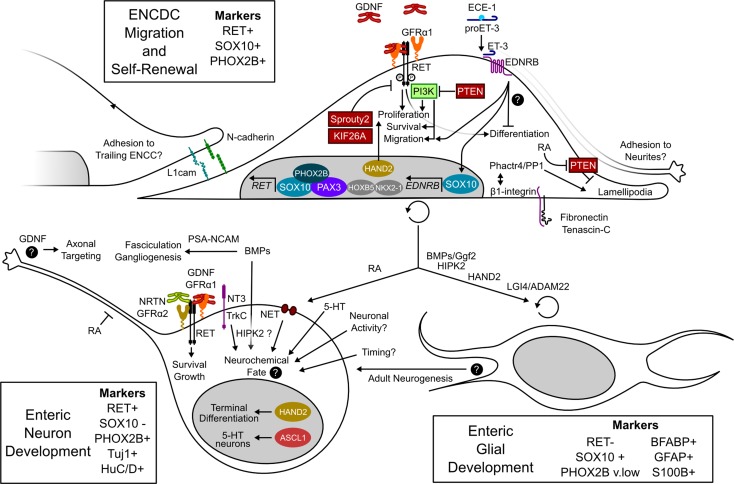
Molecules and pathways implicated in ENS development. Roles of molecules and pathways discussed in this review are shown in the contexts of ENCDC migration (*top*), neuronal differentiation (*bottom left*), and glial differentiation (*bottom right*). Markers used to distinguish these developmental stages are listed outside the cells. Intracellular signaling molecules with important activating or inhibitory roles in RET signaling within ENCDCs are boxed (inactivating in red and activating in green). Transcription factors with known (color) or likely (gray) roles in ENS development are shown in nuclei. Important mechanisms that remain unresolved, including the mechanism and targets of endothelin-3 (ET-3)/endothelin receptor type B (EDNRB) signaling in ENCDCs, the conditions that specify each subtype of neuron, the factors other than GDNF that control axonal targeting and circuit formation, and the role of neurogenesis in adults, are highlighted with black question marks. RA, retinoic acid; PSA-NCAM, polysialic acid-neural cell adhesion molecule; ECE, endothelin-converting enzyme; PP1, protein phosphatase 1; PTEN, phosphatase and tensin homolog; ENCDC, enteric neural crest-derived cell.

The trophic factors and morphogens that control ENS development depend on complex intracellular signaling pathways for their action (Fig. 3). This implicates a large number of additional proteins, the function of which in the ENS has not been directly tested and the expression patterns of which are not always restricted to the NC. For example, SHH, IHH, and GLI activity implicates important functions for the patched (PTCH1 or PTCH2) receptor and for smoothened (SMO). Similarly, BMP4 activity implies important roles for SMADs, and RA activity implies that at least some of the retinoid receptors and metabolizing enzymes [RARα, RARβ, RARγ, RXRα, RXRβ, RXRγ, RALDH1, RALDH2, RALDH3, alcohol dehydrogenase, retinol dehydrogenase, cytochrome *P*-450 (CYP) 26A, CYP26B, CYP26C, and stimulated by RA gene 6] will have essential functions that still need to be evaluated. This situation is not confined to morphogen pathways and also applies to signals downstream of critical ENS development genes. To illustrate the complexity of these signaling pathways, we briefly review the intracellular consequences of RET signaling, some of which have been directly demonstrated in ENCDCs and others inferred from non-ENCDC RET-expressing tissues and studies in cell culture.

The *RET* gene produces two protein isoforms, RET9 and RET51, which differ in their intracellular domains and have some distinct signaling properties in different cell types. After stimulation by a GDNF family ligand complexed with the appropriate GFR coreceptor, RET dimerizes and becomes autophosphorylated. Phosphorylated RET activates many intracellular signaling pathways, including phosphatidylinositol 3-kinase (PI3K) (146, 182), extracellular-regulated MAPK, JNK, p38 MAPK, PLCγ, and the small GTPase Rac (70). These pathways are activated by adapter complexes that bind to phosphorylated RET intracellular domains. c-SRC also binds directly to activated RET and contributes to PI3K activation (59, 60). Important docking tyrosines known to be required for ENS development include tyrosine 981, the docking site for SRC, and tyrosine 1015, which activates PLCγ. Tyrosine 1062 of the RET9 isoform is especially critical for ENS development (102, 211) and serves as a docking site for the adapter proteins SHC and GRB2, mediating activation of the MAPK and PI3K pathways. Negative regulators of RET are also required for normal ENS development and maturation. Mice lacking *Sprouty2*, a negative regulator of receptor tyrosine kinase signaling, have hyperganglionosis, esophageal dysmotility, and intestinal motility defects due to hypersensitivity of RET to GDNF signaling (189). Another recent study implicates KIF26A, an atypical kinesin, in the negative regulation of RET through the binding and inhibition of GRB2. Mice lacking *Kif26a* develop megacolon and hyperganglionosis and appear to have defects in neurite growth, despite an overactive GDNF/RET signaling system (226). Overactivation of RET also occurs in the context of MEN2A, which is occasionally coincident with HSCR. This paradoxical situation demonstrates that the same mutation can have activating effects (i.e., oncogenesis) in one system and inactivating effects (i.e., ENS development) in another. One possible mechanism for this is that some MEN2A mutations, which result in inappropriate intermolecular disulfide bond formation, activate RET via constitutive dimer formation but disrupt RET structure and prevent its efficient expression at the cell surface (188). Protein trafficking to the cell surface may be more efficient in some cells than in others, or perhaps the rapid rate of ENCDC division does not permit the accumulation of poorly trafficked, but hyperactive, protein.

Of the pathways activated downstream of RET, the PI3K pathway appears to be the most critical for ENCDC migration. Studies of ENCDC migration in the presence of PI3K inhibitors have demonstrated the importance of this pathway for migration toward GDNF (146). PI3K phosphorylates phosphatidylinositol (4,5)-bisphosphate to generate phosphatidylinositol (3,4,5)-trisphosphate (PIP_3_), which recruits the kinases phosphoinositide-dependent kinase 1 and AKT to the membrane. In addition to targets downstream of AKT, PIP_3_ accumulation increases the local activity of the Rho GTPases RAC1 and CDC42 through their guanine nucleotide exchange factors, contributing to cell motility and neurite extension (83). This process also recruits the partitioning defective (PAR) complex of polarity proteins (PAR3/PAR6/PKCζ), which influence axon specification and growth. PKCζ, an atypical PKC, is then activated by PIP_3_ and phosphoinositide-dependent kinase 1 and may locally inhibit glycogen synthase kinase-β (GSK3β), which must be disabled for definition and efficient growth of axons. In differentiating enteric neurons, inhibition of PKCζ or GSK3β increased the number of neurons developing multiple axons and decreased neurite growth (202). PKCζ and GSK3β inhibition also reduced ENCDC invasion of the colon in organ culture assays, suggesting a role for polarity effectors in the migration of undifferentiated ENCDCs or a role for neurite growth in the colonization process.

Molecules that inhibit the PI3K cascade are also involved in ENS development. PTEN is a tumor suppressor protein that reverses the reaction catalyzed by PI3K, preventing activation of downstream effectors. In the ENS, PTEN serves as a “brake” on ENCDC migration, proliferation, and growth. One recent study genetically ablated *Pten* within the mouse NC, which caused intestinal hyperganglionosis and megacolon. These animals also have overactivation of AKT and other downstream targets of the PI3K pathway within the ENS (162). Enteric neuron hyperplasia began at embryonic *day 16*, several days after colonization of the colon by ENCDCs. Another recent study complements these postcolonization findings, showing that PTEN levels must be reduced within migratory ENCDCs at the wave front for efficient migration (65). Furthermore, in cultured cells responding chemotactically to GDNF, PTEN was polarized away from the leading edge of the cell, and PTEN overexpression impaired ENCDC migration.

## Progress in Answering the Persistent Questions in ENS Developmental Biology

Despite dramatic advances in our understanding of the molecular and cellular mechanisms of ENS development, many important questions remain only partially addressed.

## Why Do ENS Precursors Migrate Through the Bowel?

It has been difficult to identify a master mechanism that controls the migration of ENCDCs. Clearly, the GDNF/RET/GFRα1 signaling pathway is critical for migration of ENCDCs out of explants (146, 219) and for their directional migration through Boyden chamber membranes (65). GDNF is also mitogenic to ENCDCs and, at later stages of development, trophic for differentiating enteric neurons. The expression of RET in the vagal NC begins at or before embryonic *day 9*, prior to invasion of the foregut (57). At the same time, the foregut mesenchyme begins to express *Gdnf* mRNA, so the GDNF protein can attract pre-ENCDCs adjacent to the foregut (146). In addition, *Gdnf* expression along the gut mesenchyme appears to be spatiotemporally patterned. At embryonic *day 9.5*, *Gdnf* mRNA is abundant in the stomach. By embryonic *day 10.5*, *Gdnf* mRNA extends to the cecum and is most intense in this region. At both of these time points, the ENCDC wave front is rostral to the *Gdnf* expression peak. However, the cecum sustains the highest level of *Gdnf* mRNA until ENCDCs complete their colonization of the terminal colon (146). This suggests a role for a gradient of GDNF in promoting ENCDC migration, at least up to the point where ENCDCs pass through the cecum, after which GDNF chemoattraction cannot explain their continued migration, since *Gdnf* mRNA levels are lower in more-distal bowel. GDNF is clearly chemoattractive to ENCDCs in cell and organotypic culture (65, 146, 219), but the ability of endogenous GDNF to induce directed chemotaxis of ENCDCs within the bowel mesenchyme has been difficult to demonstrate in vivo. In addition to attracting pre-ENCDCs into the foregut, long-range GDNF signals might also have an important role during the entry of the very first ENCDCs into the colon at embryonic *day 11* in the mouse. A recent study (151) has determined that most of these pioneer cells actually enter the colon by crossing the mesentery between the closely apposed midgut and hindgut (Fig. 1*C*). Unlike the majority of ENCDCs, which migrate through the bowel wall, these cells exit the midgut and migrate across the mesentery as isolated cells. This study also showed that a thin band of antimesenteric colon mesenchyme expresses *Gdnf* mRNA at this time point and that the mesenteric crossing process requires GFRα1, suggesting that a long-range gradient of GDNF attracts these ENCDCs into the colon. By combining organ culture and a mouse line expressing a photoconvertible fluorescent protein in ENCDCs, the authors were able to mark these cells and demonstrate that the ENS in the distal colon is derived almost entirely from ENCDCs that cross the mesentery (151).

A model that explains many aspects of vagal ENCDC migration within the bowel mesenchyme (170, 193) is based on the observation that NC cells migrate efficiently through the bowel only when they are at high densities and proliferating. According to this model, migration need not be directed toward a particular attractive signal at the end of the bowel. Instead, the only mechanisms required to produce a directionally migrating wave front of cells are a proliferating cell population, a limited “carrying capacity” of the local microenvironment, and random motility of ENCDCs. Proliferation in one region proceeds until a limiting cell density is reached, and then it stops. The translocation of the wave front proceeds mostly by the proliferation and random movement of cells at the wave front (181). Thus migration of individual cells need not be directional for a moving wave front to develop (180).

There is ample evidence to support this model. The first experiments demonstrating a NC origin for the enteric ganglia showed that removal of the vagal NC abolished ganglia throughout the digestive tract and that partial ablation produced partial aganglionosis, always in the distal region of the bowel (217). Indeed, mechanically reducing the numbers of ENCDCs in bowel explants reduces the population's migration speed (218) and reduces their invasion of the colon (53). ENCDC proliferation is also required for wave front advance (181). In addition, there is strong evidence that ENCDC migration is not intrinsically unidirectional through the bowel, since ENCDCs grafted at the caudal ends of aneural bowel can migrate caudorostally (181, 220), and vagal neural tube grafted into the sacral level of the neuraxis of chick embryos results in ENCDCs that efficiently migrate caudorostrally through the bowel (27). Finally, it is likely that the bowel microenvironment has a limited carrying capacity for ENCDCs. Even in the absence of any other limiting factors, availability of GDNF limits the proliferation of ENCDCs above a maximal density (80, 208).

The proliferation-dependent model cannot explain all aspects of ENCDC colonization. According to simulations based on this model, purely random diffusion would be sufficient to create a migrating wave front (180), but observations of migrating ENCDCs demonstrate complex and nonrandom patterns of movement (Fig. 2*A*). ENCDCs migrate in contact with one another in structures that, near the wave front, resemble caudally projecting “chains” of cells. Time-lapse imaging of fluorescent ENCDCs in organ culture reveals that the ENCDCs in these chains climb on each other and have unpredictable trajectories (218). ENCDCs can detach from chains, sometimes forming new chains, or can advance along an existing chain (54). However, the overall structure of the chains and the spaces between them are persistent over time, despite the dynamic behavior of each ENCDC. These complex behaviors strongly suggest additional signals governing ENCDC guidance that remain to be discovered. Furthermore, early neuronal differentiation begins almost immediately behind the wave front, and neurites grow along chains of ENCDCs. The nascent neuronal cell bodies also migrate along these neurites, which generally project rostrocaudally (86, 220). At the wave front itself, migration trajectories of ENCDCs are also predominantly caudal (151, 218), suggesting that wave front ENCDCs migrate toward a local cue. It is possible that the ENCDC population generates a gradient of GDNF by consuming or competing for GDNF. Endocytosis or simply receptor binding of GDNF by ENCDCs may deplete most of the available GDNF behind the wave front, creating a local gradient that travels with the wave front.

Another phenomenon that might contribute to the directed migration of wave front cells is contact inhibition of locomotion. Recent experiments in *Xenopus* NC demonstrated that directional migration of NC cells is inhibited by contact with other NC cells, but not with other cell types (48a). Furthermore, De Calisto et al. (48a) found that noncanonical Wnt signaling (planar cell polarity) at cell-cell contacts mediates this repulsion. Disruption of this pathway inhibits directional migration (34). This pathway, in turn, is dependent on the function of primary cilia on NC cells and is disturbed in Bardet-Beidel syndrome, an HSCR-associated condition caused by ciliary gene dysfunction (168). The caudally directed migration of individual ENCDCs at the wave front could be driven by such a mechanism. Some aspects of colonization, such as the failure of vagal ENCDCs to colonize already colonized bowel, might be explained by this behavior, but other aspects, such as chain migration, seem incompatible with this mechanism.

The proliferation-dependent and contact-mediated repulsion models of migration may explain some otherwise perplexing non-cell-autonomous effects of ENS gene mutations. Mouse chimera and grafting experiments have shown that mixing migration-capable NC cells with a sufficient number of NC cells with genetic lesions in *Ret* (16), *Ednrb* (107), or *Sox10* (106) impairs migration of wild-type ENCDCs enough to cause distal aganglionosis in chimeric embryos and grafted bowel tissue. Wild-type ENCDCs were also able to rescue the migration of *Ednrb-*null mutant ENCDCs in some chimeric embryos. Since RET, EDNRB, and SOX10 are primarily expressed within ENCDCs, the non-cell-autonomous effects exerted on neighboring normal ENCDCs are surprising. These results are consistent with a proliferation-dependent model of migration, which predicts that if some part of the migratory population is defective for proliferation or survival, as is the case in these models, incompetent cells at the leading edge of the wave front can inhibit the progress of the cells in the more-proximal bowel by reducing the size of the proliferating cell population and blocking progress forward. Similarly, the ability of normal ENCDCs to rescue the migration of mutant ENCDCs may be rooted in an increased overall population size or proliferative capacity. However, it may also occur because wild-type cells actively invade aganglionic bowel, forming a substrate for mutant cells to migrate via chain migration or migration along neurites.

Several processes, including some that fit a proliferation model (wave front movement), some that appear more chemotactic (movement of individual cells at the wave front), and some related to cell or matrix adhesion (fiber climbing and chain migration), occur simultaneously during colonization of the bowel. Determining experimentally which of these processes are critical for colonization has been difficult, largely because mechanisms responsible for directed migration, proliferation, and neurite growth share many molecules and pathways, making any experimental separation difficult. Despite this difficulty, roles for several molecules involved in neuronal polarity and cell motility have been demonstrated. As discussed previously, inhibition of the neuronal polarity effectors GSK3β and PKCζ impairs ENCDC migration (202). Also, chemical inhibition of the RAC and CDC42 GTPases or the RHO effectors ROCKI and ROCKII in the cecum and colon reduced migration and neurite growth without affecting proliferation (185). In another study, genetic ablation of *Rac1* and *Cdc42* in the early NC impaired NC cell proliferation and, thereby, prevented colonization of the distal bowel by ENCDCs but did not cause migration defects in early NC cells emigrating from the neural tube (69). While both studies implicate Rho family GTPases in ENS development, they suggest different roles of these molecules in different stages of NC development.

Finally, we should not neglect the critical role of the ECM and the ENCDC proteins that interact with the ECM during bowel colonization. The ECM provides a mechanical substrate and important signals for ENCDC migration and differentiation. During ENCDC migration, the bowel mesenchyme matures from a uniform-appearing population of mesenchymal cells into layers with distinct morphologies and ECM molecule expression patterns (147). Maturation occurs in a bidirectional wave from rostral and caudal ends of the bowel and more quickly than ENCDC colonization, so the ECM in contact with ENCDCs is constantly changing. Laminin influences axon guidance, as noted previously, and also enhances neuronal differentiation (43). Since newly differentiated neurons migrate more slowly than undifferentiated ENCDCs (86), the high levels of laminin in the colon may contribute to distal bowel aganglionosis.

Important roles have also been assigned to several ECM-interacting molecules. β_1_-Integrin (*Itgb1*) is important for ENCDC migration, and its loss from ENCDCs results in colonic aganglionosis and structural abnormalities of the ENS (24). Integrins are cell surface receptors for ECM molecules that participate in adhesion and signaling. β_1_-Integrins are necessary for optimal ENCDC migration on fibronectin, which is present throughout the bowel and is enriched in the hindgut. β_1_-Integrin is especially critical for migration on the ECM molecule tenascin-C, which is expressed at high levels in the hindgut and otherwise inhibits ENCDC migration (21). β_1_-Integrin is also important for transducing signals from the ECM, and dysregulation of these signals impairs ENS development. PHACTR4, a protein recently shown to be required for directed ENCDC migration, interacts with the actin cytoskeleton and protein phosphatase 1 (PP1). PHACTR4, through its interaction with PP1, modulates β_1_-integrin signaling and activates the actin-severing protein cofilin, contributing to the formation of directionally stable lamellipodia (225). This manifests as hypoganglionosis in *Phactr4*^*humdy/humdy*^ mutant mice, which lack interaction between PHACTR4 and PP1. During development, these embryos have less directed ENCDC migration at the wave front, despite having a normal random migration velocity.

Adhesion between an individual ENCDC and other ENCDCs is also important for migration. The homophilic adhesion molecules N-cadherin, NCAM, and L1 cell adhesion molecule (L1CAM) are expressed by migrating ENCDCs, and loss of N-cadherin or L1CAM results in delayed ENCDC migration and potentiates aganglionosis (5, 24, 206), although neither is sufficient to cause aganglionosis by itself. Finally, as previously discussed in the context of BMP signaling, the posttranslational addition of polysialic acid to NCAM influences ENCDC aggregation and migration efficiency (62, 67).

Migration and proliferation differ significantly between ENCDCs derived from the vagal and sacral NC. Sacral crest-derived ENCDCs migrate in isolation, rather than in chains (209), always moving along extrinsic neuronal fibers that project into the hindgut. Although sacral ENCDCs arrive in the terminal hindgut at the same time as vagal ENCDCs, they continue to migrate caudorostrally through vagal crest-colonized bowel. In contrast, vagal ENCDCs will not enter previously colonized bowel (97). The proliferative capacity of sacral ENCDCs is also very different from that of vagal ENCDCs. While sacral ENCDCs normally comprise ∼10–20% of the most-distal region of the ENS (28, 209), their population only slightly expands if the vagal NC is mechanically ablated in chick embryos (26) and the hindgut is otherwise devoid of ENCDCs. Vagal-to-sacral transplantation experiments in the chick (27) have demonstrated that at least some of these differences in behavior reflect intrinsic differences between sacral and vagal NC, rather than different signals along the migration routes. While vagal and sacral ENCDCs express the same ENCDC-specific markers (3, 49), a RNA microarray comparing vagal and sacral chick neural tube explants (49) indicated that sacral-derived crest expressed less RET mRNA than vagal crest, and their behavior was partially transformed to that of vagal crest by RET overexpression. A study in mice where the vagal wave front was significantly delayed (NC-specific *Ednrb* deletion) confirmed the finding that RET expression was reduced in sacral ENCDCs (61). Notably, in the *Ret*^−/−^ and *Gfra1*^−/−^ mouse models of total intestinal aganglionosis (57, 30) and a conditional *Ednrb* ablation model of colonic aganglionosis (61), rare intrinsic neurons can be found within the most-distal bowel. These almost certainly represent the remnants of the sacral ENCDC population. Since these neurons are rare and their numbers approach neither the expected sacral-derived densities observed in the chick nor those estimated in mouse organ culture experiments (28, 209), it seems likely that mutations affecting vagal ENCDCs also affect sacral ENCDCs, which likely accounts for their absence in the terminal colon of HSCR patients. To our knowledge, it is not known whether the aganglionic segment of HSCR-affected terminal colon contains any residual neurons, but they would likely be difficult to detect using routine diagnostic histology and would not form a functional ENS.

## What Is the Function of EDNRB in ENS Development?

Although EDNRB/EDN3 signaling is essential for efficient colonization of the colon and clearly influences ENCDC differentiation and migration, many of the cellular and molecular effects of EDNRB/EDN3 signaling on the developing ENS remain confusing. Total loss of EDNRB signaling results in colonic aganglionosis, abnormalities of the ENS in the small bowel (33), and developmental delay of ENCDC migration (55). This is a much milder phenotype than is associated with loss of RET signaling. Two primary processes within ENCDCs are affected by EDNRB: *1*) EDNRB prevents premature neuronal differentiation; and *2*) EDNRB is required for efficient migration within the colon. Here we summarize the well-established actions of EDNRB and discuss some apparently contradictory observations demonstrating species-specific, region-specific, and cell type-specific roles for EDNRB in the ENS.

In culture, there is significant evidence that the EDNRB ligand ET-3 maintains ENCDCs in an undifferentiated state (19, 92, 213). ET-3 alone does not appear to cause proliferation, but ET-3 treatment causes overpopulation of the developing ENS in avian gut explants (143) and acts, together with GDNF, to increase the proliferation of undifferentiated mouse ENCDCs (8). ET-3 administration to cultured enteric progenitors maintains their expression of SOX10 and their undifferentiated state (19), suggesting that EDNRB signaling might be required to prevent premature loss of SOX10 protein. These observations are consistent with a role for ET-3/EDNRB signaling in repressing neuronal differentiation, which might otherwise be triggered by the high GDNF levels in the cecum and rising laminin levels in the colon (55). Since neurons are postmitotic, enhanced neuronal differentiation will reduce the proliferative drive that supports bowel colonization by ENCDCs. The similarity of the phenotypes that result from *EDNRB* or *SOX10* mutations (colonic aganglionosis) and their genetic interaction when mutated (see below) further suggest that EDNRB and SOX10 are components of a common pathway that keeps ENCDCs undifferentiated. In mouse ENCDCs, *Ednrb* expression is directly regulated by SOX10 binding to promoter elements upstream of *Ednrb* (228), possibly forming a positive-feedback loop contingent on ET-3 signaling.

It is unclear whether increased neuronal differentiation occurs in vivo when EDNRB signaling is defective. The best evidence, from homozygous-null *Edn3* mouse embryos, showed an increase in the percentage of wave front ENCDCs positive for neuron-specific β_III_-tubulin, indicating an increase in early neuronal differentiation (19). However, in a recent study that used a conditional allele of *Ednrb* allowing specific ablation from the NC, the wave front did not display an increased proportion of ENCDCs positive for the neuronal marker Hu (55). In another study of *Ednrb-*null rat embryos, the wave front also failed to display an increase in peripherin-positive cells (116). This same study showed that rat enteric NC stem cells (NCSCs), a defined subpopulation of crest-derived cells in the gut, respond to ET-3 in culture by differentiating into myofibroblast-like cells. It is unclear whether differences between cell types, the mutation status of *Edn3* or *Ednrb*, the neuronal markers chosen, or the species studied account for these differing results, suggesting the need for additional investigation of the role of EDNRB signaling in the ENS.

Despite the fact that ENCDCs lacking *Ednrb* have a migratory delay throughout ENS development, ENCDCs have a specific requirement for EDNRB signaling as they migrate through the colon. In grafting experiments performed in organotypic culture, NC cells from normal bowel colonized normal embryonic colon but did not invade embryonic colon from mice with *Edn3* mutations (101). *Edn3* mRNA appears to be expressed in a spatially and temporally regulated manner that tracks the migration of ENCDCs, and EDNRB signaling is required during a very narrow temporal window roughly corresponding to colonic migration. At embryonic *day 10*, *Edn3* mRNA is expressed throughout the midgut, but levels become elevated in the cecum at embryonic *day 10.5*, and this domain of expression extends into the hindgut at embryonic *day 11*, when ENCDCs are migrating through the cecum (8). This is identical to the temporal interval when EDNRB signaling is required for ENS development (embryonic *days 10.5–12.5*), as shown using a tetracycline-regulated *Ednrb* knockin mouse (178). Similar to the effects on differentiation, some of the effects of ET-3/EDNRB signaling on migration are also contradictory and difficult to interpret. One issue is that ET-3 appears to have divergent effects on ENCDC migration under different conditions. ET-3 impairs GDNF's chemoattractive effects on ENCDCs in explants cultured in collagen gels (8, 116, 143) but appears to encourage migration through the colon in explant cultures (143) and to partially rescue colon colonization when RET signaling is dysfunctional (203). EDNRB antagonists also cause colonic hypoganglionosis or aganglionosis in culture (143, 212), and acute chemical inhibition of EDNRB in colonic ENCDCs produces immediate retraction of cell processes and loss of motility, which occur too quickly to result from effects on differentiation (55). In addition to its expression in ENCDCs, EDNRB is expressed to some degree in the mesenchyme (8) of mouse bowel. This observation, combined with the finding that laminin-α expression by enteric smooth muscle cells is negatively regulated by ET-3 (213), suggested that EDNRB signaling in the mesenchyme might be necessary to create a colonic microenvironment permissive to ENCDC colonization. However, mesenchymal expression of EDNRB is not conserved in the chick (143, 144). Furthermore, NC-specific ablation of *Ednrb* produces the same ENS phenotype as a null allele (56), and colonic aganglionosis in rats lacking *Ednrb* can be rescued with a transgene that expresses functional EDNRB specifically in ENCDCs (78), indicating that NC cells are the critical targets of the ET-3 signaling required for ENS development.

The importance of each second messenger pathway activated downstream of EDNRB is also unclear. One disease-causing mutation in *EDNRB* has been linked to a selective loss of Gα_q_/Gα_11_ coupling and intracellular Ca^2+^ signaling (100, 161), while two others have been shown to perturb Gα_i_ coupling and prevent the reduction in cAMP levels that occurs with Gα_i_ activation (68). Of these possibilities, there is more evidence for EDNRB signaling through cAMP in ENCDCs. Indeed, NC-restricted deletion of Gα_q_/Gα_11_ did not result in any ENS defects (51). In primary enteric progenitor cell culture, the antidifferentiation actions of ET-3 were mimicked by inhibition of the cAMP-regulated PKA and suppressed by increasing cAMP (8). In the same study, a PKC inhibitor did not appear to inhibit the effects of EDNRB stimulation, which would be likely if some of the actions of EDNRB were mediated through Gα_q_/Gα_11_. Interestingly, cAMP-dependent activation and cAMP-independent activation of PKA downstream of BMP signaling have important roles controlling differentiation of another NC-derived population, noradrenergic sympathetic neurons (129). However, inhibition of PKA activity is probably not uniformly beneficial to the developing ENS. PKA has been shown to phosphorylate RET at a serine residue (70) important for lamellipodia formation in culture. Targeted mutation of this site to prevent phosphorylation results in distal colonic aganglionosis and ENCDC migration defects (6). Moreover, the same study demonstrated that PKA inhibition reduced ENCDC migration in the colon. This requirement of PKA activity for migration is difficult to reconcile with the evidence for inhibition of cAMP signaling required to maintain ENCDCs in an undifferentiated state. Intermediate levels of PKA activation or fine temporal or spatial control of cAMP or PKA may be required for normal ENS development. Further study of the ET-3/EDNRB signaling pathway in ENCDCs is necessary for a better understanding of these important molecules.

## What Controls Neuronal vs. Glial Differentiation of ENS Precursors, and What Controls Neuronal Subtype Specification?

Although appropriate differentiation into the many neuronal classes and into glia is absolutely critical for ENS function, the signals that control these cell fate decisions are less well understood than the process of initial ENS colonization by the multipotent ENCDCs.

## ENCDC, Neuron, or Glial Cell?

Presumably, an ENCDC must decide whether to self-renew or differentiate into a neuronal or a glial progenitor. While SOX10 has a central role in maintaining ENCDCs (19, 112), it is not sufficient for maintaining an undifferentiated state, since ENCDCs and adult enteric glia express SOX10. Notch signaling is implicated in gliogenesis in other areas of the PNS but appears to have a different role in the ENS. As newborns, mice with NC incapable of receiving Notch signals develop a hypocellular ENS, accompanied by reduced Sox10 expression in migrating ENCDCs and an inappropriately high level of neuronal differentiation in the population of migrating ENCDCs. Thus, in the developing ENS, Notch is required to prevent premature neuronal differentiation and depletion of undifferentiated ENCDCs. One signal recently demonstrated to be important for enteric glial development is the secreted factor LGI4, which is produced by migrating ENCDCs in the bowel and glia themselves. Mutations in *Lgi4* or its receptor *ADAM22* reduce the number of enteric glia and alter ENS structure (150). BMP signaling may be involved in specifying enteric glia, since it induces glial differentiation of ENCDCs in vitro, and these developing glia become dependent on glial growth factor 2 (Ggf2, a NRG1 isoform) signaling through ErbB3 for survival (39). RA signaling also increases neuronal differentiation and the proliferation of cells with early neuronal markers, but not at the expense of glia (174).

### Neurogenesis in the ENS is asynchronous.

Cells expressing early neuronal markers (Tubb3/Tuj1 and HuC/HuD) and bearing long processes appear in the ENS almost immediately after colonization begins (86, 221). A core population remains as undifferentiated ENCDCs and is presumably responsible for propagating the ENCDC wave front down the bowel. Other cells express pan-neuronal markers and extend neurites but remain in the cell cycle and continue to migrate. Still others exit the cell cycle during specific intervals (a neuron's “birth date”) and differentiate into diverse enteric neuron subtypes. Neuronal birth dating is a technique that exploits labels, such as bromodeoxyuridine or tritiated thymidine, that are permanently integrated into the DNA of replicating cells. The label is administered at one selected time point, and development is allowed to continue. Neuronal precursors that incorporate the label and then become postmitotic retain high levels of the label, while cells that continue to divide dilute the label to undetectable levels. Thus this technique marks cells preparing for their final division. In the mouse, serotonergic neurons are born earliest (embryonic *days 9–15*), and birth of cholinergic neurons peaks at embryonic *day 14* and continues until embryonic *day 17*. Birth dates for dopaminergic, peptidergic, nitrergic, and GABAergic neurons peak at embryonic *day 14* in the myenteric plexus and close to birth (postnatal *day 0*) in the submucosal plexus, extending into postnatal life for up to 2 wk after birth (41, 156). While the expression of a neurochemical phenotype occurs sometime after a given neuron's birth, the tight association between the time of cell cycle exit and neurochemical phenotypes suggests that the timing of cell cycle exit may control some aspect of neurochemical fate. Alternatively, an upstream mechanism that remains unknown may determine birth date and ultimate fate.

### Signals and genes affecting neuronal subtypes.

There are relatively few genetic models that lack subsets of enteric neurons. This is likely due to the difficulty of identifying subtle ENS phenotypes, which may not lead to life-threatening bowel dysfunction. A prominent exception is serotonin (5-HT)-producing neurons, which are absolutely dependent on the transcription factor ASCL1. Serotonergic and, to a lesser extent, calretinin-expressing neurons also require the norepinephrine transporter (*Slc6a2*) to develop in proper numbers (125). Another transcription factor required for terminal differentiation of enteric neurons is HAND2, a basic helix-loop-helix transcription factor needed for heart and NC development. *Hand2* is not required for ENCDC migration down the bowel, but its deletion results in profound defects in overall ENS structure (93), reductions in neuronal density (93, 48), severe bowel distension likely caused by ENS defects (123), and subtype-selective (93, 123) or a more general (48) failure to differentiate into functional neurons. Overexpression studies demonstrate that HAND2 is sufficient to support neurogenesis (93) and specify VIP expression in cultured chick ENCDCs, while early NC-specific deletion of mouse *Hand2* results in a loss of VIP-expressing neurons (93). In a mouse model where *Hand2* is deleted in a specific subset of ENCDCs (123), precursor proliferation, gliogenesis, and the specification of many (cholinergic, nitrinergic, and calretinin-expressing), but not all, neuronal subtypes were impaired within the population derived from *Hand2-*deleted ENCDCs. Reductions in numbers of specific neuronal subtypes (nitrergic and calretinin-expressing, but not substance P-expressing) also result from haploinsufficiency for and hypomorphic alleles of *Hand2* (47). Since *Hand2* is expressed and experimentally deleted in undifferentiated ENCDCs and differentiating neurons and glia, the precise stage where loss of *Hand2* alters neuronal subtype specification or gliogenesis is not known.

Another signal critical for specific cell populations in the ENS is neurotrophin-3 (NT-3), which signals through the p75 neurotrophin receptor and the TrkC receptor. Mice lacking NT-3 or TrkC had significantly fewer neurons throughout the ENS, with a particular deficit in the submucosal plexus (42). Calcitonin gene-related peptide-reactive submucosal neurons are most sensitive to loss of NT-3/TrkC signaling. However, this signaling pathway does not appear to uniquely identify a single type of neuron. BMP2 and BMP4 signaling also influence neuronal subtype and enhance the development of this TrkC-positive population (38, 41). When the BMP inhibitor Noggin was expressed ectopically in all enteric neurons in vivo, the overall neuronal density in the ENS increased markedly, but the number and proportion of TrkC-positive neurons were reduced. Conversely, a transgene expressing BMP4 increased the proportion of TrkC-positive neurons in the adult ENS. These studies implicate BMP signaling in specification of a particular neuronal subtype (TrkC-positive) and limitation of the numbers of other neurons (TrkC-negative). In particular, the density and proportion of early-born classes of neuron were increased, and the density and proportion of late-born classes were decreased, when BMP signaling was inhibited by the Noggin transgene (41). In accordance with the effects of BMPs on glial differentiation in culture, the same Noggin transgene also reduced the density and proportion of glia in the ENS (39).

### Early neuronal activity shapes late-differentiating neurons.

Since enteric neurons are “born” asynchronously, the activity of early-born neurons has the potential to shape developmental decisions in later-born neuronal populations. 5-HT-producing neurons are among the earliest-born populations in the ENS (156), and the 5-HT produced by these cells has a significant effect on the development of later-born neuronal populations (126). ENCDCs express many classes of 5-HT receptors, and 5-HT promotes the neuronal differentiation of ENCDCs in culture. Mice with enteric neurons unable to synthesize 5-HT develop fewer neurons of several late-born classes, including dopaminergic, GABAergic, and a subset of nitrergic neurons. Serotonergic neurons, in turn, require the norepinephrine transporter for proper development, which suggests that norepinephrine uptake may shape neuronal differentiation. Recent work has also demonstrated that nascent enteric neurons are electrically active very early in the colonization process (87) and that inhibition of this activity reduces the number of early-born nitrergic neurons close to the ENCDC wave front (88), suggesting that the interdependence of different types of enteric neuron is not limited to the late-born populations. Finally, neuronal activity may be important for ENCDC colonization of the bowel, since tetanus and botulinum neurotoxins slow neurite extension and ENCDC migration (201).

### Lineage restriction and decision points.

There are a few known progenitor states that mark major decision points in the enteric neuron generation program. One important mark of lineage restriction is the transiently catecholaminergic (TC) class of immature enteric neuron. Neurons expressing catecholaminergic markers are common early in the colonization process, but definitive catecholaminergic neurons represent a small fraction of the mature ENS. The early, TC lineage encompasses many terminal fates, but it includes all future serotonergic neurons (7) and excludes certain fates, including late-born calcitonin gene-related peptide-producing neurons (15). TC status is clearly a marker of an important decision-making step, but the factors controlling this decision remain unknown, although *Ascl1* may contribute, since it is required for development of the TC lineage (15). Also, since TC cells produce norepinephrine and TC cells and the TC-derived lineage express the norepinephrine transporter, the TC lineage may influence its own developmental fate by signaling through norepinephrine.

## What Controls Neurite Outgrowth and Axon Pathfinding in the ENS?

The ENS is controlled by the organized connections between neurons of different types in different regions. In the CNS and other regions of the PNS, target-derived trophic factors ensure that specific neuronal subtypes are matched qualitatively and quantitatively to their targets. This system works well, because axon tips and neuronal cell bodies are usually distant from each other and in quite distinct environments. In contrast, ENS neurons often have a similar environment at the axon tip and cell body (e.g., for cells whose soma and neurites remain within the myenteric plexus), making it difficult to imagine how target-derived trophic factors might direct the proper wiring of the adult ENS. While distribution of neuronal classes clearly differs between different areas of the ENS, each ganglion is indistinguishable from its immediate oral or aboral neighbor. Few conditions leading to defects in targeting of neuronal projections have been demonstrated in the ENS, at least in part because there has, until recently, been no simple way to track neurites of single enteric neurons. One study demonstrated that the targeting of projections from myenteric nitrergic neurons is controlled by GDNF during perinatal and postnatal development. When GDNF was ectopically expressed in enteric glia using the glial fibrillary acid protein (GFAP) promoter, NADPH diaphorase-positive (a marker of nitric oxide synthase) fibers redistributed densely around enteric glia, suggesting a role for GDNF in axon targeting for this subtype of enteric neuron (208). In contrast, neither cholinergic nor serotonergic neuron fibers redistributed toward enteric glia in these GDNF-overexpressing mice. Sasselli et al. (173) used ligand-regulated Cre recombinase and a fluorescent recombination reporter to label single enteric neurons in fetal bowel. Using this system, they were able to detect very subtle structural ENS defects and demonstrated that enteric neurons require the planar cell polarity signaling components *Celsr3* and *Fzd3* for proper wiring. Further study and innovative methods are required for a better understanding of the maturation of the nascent ENS into functional circuits.

## What Is the Normal Role of Cell Death in the Developing and Mature ENS?

Programmed cell death in the form of apoptosis plays a critical role in matching neuron numbers to target size and ensuring correct targeting of neurites in the developing vertebrate CNS and other regions of the PNS. In most regions of the nervous system, more than half of the neurons generated undergo apoptosis, often after target innervation (25). In contrast, during normal ENS development, some apoptosis occurs in pre-ENCDCs (i.e., before these cells enter the bowel), and this may be important for limiting ENS density in the proximal bowel (205). However, after ENCDC entry into the bowel, programmed cell death in the form of apoptosis does not appear to play a role in ENS development in wild-type mice. In contrast, cell death does occur in the ENS of mice with specific gene defects. In support of this statement, activated caspase-3 (a marker of cells undergoing apoptosis) is extremely rare in the fetal, newborn, and adult ENS (80) of wild-type mice. Rare instances of nuclear fragmentation and death have been observed in migrating SOX10-positive ENCDCs (45), although these events are so infrequent that they are unlikely to influence the size of the ENCDC population. Moreover, *Bax*^−/−^ and *Bid*^−/−^ mice, which have defective apoptosis in other developing neuronal populations, have an essentially normal ENS (80). Thus, programmed cell death appears to be involved in regulating the number of ENCDC precursors that initially arrive in the bowel but seems unlikely to control later developmental processes such as neuronal subtype ratios and ENS wiring. However, ENCDC apoptosis is a critical consequence of complete *Ret* and *Sox10* deficiency. Furthermore, under certain circumstances, an unusual form of cell death occurs in the ENS. When GFRα1 is genetically ablated after ENCDC migration is complete, neurons undergo nonapoptotic cell death and do not display ultrastructural signs of necrosis or autophagy (198). A subsequent study also demonstrated that a similar atypical ENCDC death occurs in the colons of mice with reduced *Ret* expression (*Ret*^9/−^) (199), a model that very closely resembles *RET-*mediated HSCR in humans. Thus this atypical cell death may prove to be a critical contributor to the most common form of HSCR.

## Applying Our Understanding of ENS Development to Human Disease

These exciting advances in our understanding of the mechanisms of ENS development raise new hope that novel strategies can be developed to reduce the frequency and severity of human intestinal motility disorders. Managing HSCR remains a challenge in the modern era. Despite advances in surgical treatment and postoperative management, 1–10% of children with HSCR die (2, 159). Furthermore, long-segment aganglionosis can necessitate the removal of enough small bowel to cause short-gut syndrome, resulting in long-term dependence on parenteral nutrition, which has serious risks of infection and liver damage. Several other less well understood clinical conditions are caused by altered ENS activity. For example, chronic idiopathic intestinal pseudoobstruction, in which intestinal motility is abnormal but neurons are present, can also be caused by structural and functional ENS defects that may or may not be obvious on routine clinical biopsies. On the basis of murine models where the ENS is formed but the bowel does not function properly, pseudoobstruction of neuronal origin is likely to be due to a variety of failures in postcolonization ENS development, such as neurotransmitter selection, axonal targeting, or synaptogenesis. For example, *RET-*activating mutations that cause MEN2B and mutations in *FLNA* (77) cause dysmotility, but the motility defects remain incompletely characterized. In contrast to the major defects that may underlie chronic pseudoobstruction, even more subtle changes to the physiology of the ENS may contribute to irritable bowel syndrome and other “functional” motility disorders. In fact, genetic lesions that alter the structure of the ENS can produce or modify bowel inflammation, suggesting that developmental abnormalities of the ENS can contribute to the severity of inflammatory bowel disease (29, 134). Understanding neuronal cell fate decisions and the wiring process that generates the normal ENS will help us better understand how these pathophysiological events impair intestinal function and may suggest novel clinical interventions for intestinal motility and inflammatory diseases.

## Why Is HSCR Partially Penetrant, and Why Does the Extent of Aganglionosis Vary Between Individuals?

Human birth defects, including HSCR, result from genetic defects, nongenetic factors, or interactions between genes and “fetal environmental” factors. In some cases, single-gene defects are the major risk factor for HSCR occurrence and have very high penetrance. However, no known HSCR-associated gene defect is fully penetrant. A partial explanation for this observation is that genetic interactions critically influence HSCR penetrance. For example, there is a well-established genetic interaction between *EDNRB* and *RET* mutations in humans and in mice. Alleles of each gene that produce mild phenotypes or no phenotype in isolation can cause severe disease in compound heterozygous mice and humans (36, 136). In mice, nonpenetrant and weakly penetrant alleles of *Ednrb* (or *Edn3*) can also worsen the severity of the ENS phenotype resulting from *Sox10* mutations (33, 183). In addition to RET coding mutations, a common intron 1 polymorphism that reduces *RET* expression (RET +3, or rs2435357) is highly associated with sporadic HSCR and modifies the penetrance of HSCR in various predisposing syndromes (58, 152, 49a). Recent studies have also implicated neuregulin 1 (*NRG1*) as a modifier of *RET*-dependent HSCR risk (76). Additionally, genes at several other chromosomal loci may influence HSCR risk in individuals with *RET* mutations (17, 72, 74, 190), although identification of the specific genes has been challenging.

Genetic interactions cannot explain all the variability of HSCR, since HSCR-like phenotypes in many inbred animal models are partially penetrant and of variable severity. *Sox10*^*Dom*^ is an excellent example of this phenomenon (33). “Developmental noise” or random occurrences at the level of individual ENCDC movement might influence migration processivity and speed. This could be translated into a variable extent of aganglionosis as the bowel wall eventually becomes relatively nonpermissive to continued invasion (55, 97) after embryonic *day 14* and the migration wave front is frozen in position, forming the transition zone between ganglionic and aganglionic bowel. The nonpermissiveness of older bowel is relative, rather than absolute, as illustrated by *Tcof1* (Treacher Collins-Franceschetti syndrome 1) mutant mice, where heterozygous mice do not fully colonize the bowel at embryonic *day 14.5* because of depletion of early NC precursors but continue to migrate, fully colonizing the colon by embryonic *day 18.5* (9). *Tcof1* ENCDCs, however, have abnormally low rates of differentiation and may, in fact, be more capable of migrating through older bowel than are wild-type ENCDCs. Another situation where ENCDCs complete their migration, despite a significant colonization delay, occurs in the rescued *Ret*^9/−^ mouse model. These mice develop colonic aganglionosis after a moderate ENCDC migration delay and ENCDC death in the colon (199), but in mice that also overexpress the prosurvival protein Bcl-XL, the colon is eventually fully colonized, even though ENCDC migration is not rescued and is incomplete at embryonic *day 13.5*. In these situations, it seems that abnormal “hardier” ENCDCs are capable of compensating for a developmental delay that would normally contribute to aganglionosis (197).

## Why Is HSCR More Common in Males than in Females?

Another perplexing issue in the study of HSCR and ENS development is the male bias for penetrant disease. Interestingly, the male bias is much more pronounced in patients with short-segment disease (5.5:1) than in those with longer regions of aganglionosis (1.75:1) (2). Conceptually, this makes sense if we consider male sex as a mild predisposing factor for aganglionosis. Among syndromic HSCR cases, length of aganglionosis mostly correlates with penetrance of a mutation (49a), and strongly penetrant mutations are not dependent on a weak modifier like sex. The molecular basis of this sex bias has been difficult to determine, despite several genetic models of colonic aganglionosis that demonstrate a similar predominance of affected males (33, 136, 199). Mutations in one X-linked gene, *L1CAM*, are rarely associated with HSCR and a group of syndromes involving multiple nervous system abnormalities and hydrocephalus. Murine studies have demonstrated that null mutations in *L1cam* can interact with *Sox10* mutations to increase the penetrance of aganglionosis and result in more severe pathology (206). Since *L1CAM* mutations cause syndromic disease, they are unlikely to account for the male predominance in isolated HSCR, unless a new and much less severe variant is found to be associated with HSCR. Another possible explanation for the male bias is suggested by the lower levels of colonic *Edn3* and *Ece1* expression in males than females during the time that ENCDCs colonize distal bowel (203). However, the reasons for this difference remain unclear, as neither testosterone nor Müllerinan inhibitory factor had a measurable effect on ENCDC migration or gene expression. Addition of ET-3 to cultured Ret mutant male mouse bowel, however, increased the extent of colonization in vitro, suggesting that EDNRB signaling is limiting in male mice. Clarifying the mechanisms behind these sex differences will require a better understanding of sexual dimorphism at the level of gene expression, with more detailed analysis of *cis*- and *trans*-regulatory elements (e.g., for *Edn3* and *Ece1*) and the epigenetic marks that control gene expression for critical regulators of ENS development.

## Why Does Down Syndrome Predispose to HSCR?

Down syndrome (trisomy 21) is the most common genetic disorder that predisposes to HSCR. Overall occurrence of HSCR in Down syndrome is low (∼1%), and the common RET +3 polymorphism is highly associated with HSCR among children with Down syndrome, suggesting that some level of *RET* dysfunction is required for penetrant disease (49a). Although HSCR occurs in Down syndrome with a low penetrance relative to single-gene syndromes such as WS4 and Mowat-Wilson syndrome, Down syndrome contributes to 2–10% of HSCR cases (2) because it is quite common (∼1 in 800 births). Increased chromosomal copy number of genes expressed in the ENS or surrounding tissues could be important for the HSCR-predisposing effect of trisomy 21. However, no genes confirmed to be important to ENS development reside on chromosome 21, although some candidates have been identified (135). One of these is *DSCAM*, an immunoglobulin-superfamily cell adhesion molecule expressed widely in the CNS and the developing ENS (214). A high-resolution copy number study of individuals with partial trisomy 21 and birth defects including HSCR demonstrated a shared 13-megabase region containing *DSCAM* that was duplicated in the three study participants with HSCR (113). It will be interesting to see whether *DSCAM* or other genes from this critical region impair ENS development if overexpressed.

## Stem Cells in the ENS: Therapeutic Possibilities and Natural Roles

During ENS colonization, ENCDCs serve as stem cells for the ENS and engage in self-renewing replication and terminal differentiation into neurons and glia. Similar cells exist in the adult and newborn bowel in humans and rodents (for review see Refs. 90 and 98). Understanding these cells is critical for any future attempts to use them in therapy for HSCR, gastroparesis, achalasia, intestinal pseudoobstruction syndrome, or possibly CNS disorders. Some stem cell types that have been transplanted into the rodent bowel are not NC derivatives but, instead, begin as embryonic stem cells (99) or CNS neural stem cells, which can improve gastric emptying in a mouse model of gastroparesis (141) when transplanted into the pylorus. However, we focus our discussion on stem cells derived from the ENS.

Cultures of multipotent and self-renewing enteric neurospheres can be established from embryonic and postnatal mouse bowel (20, 186). Human enteric neurospheres have also been grown from full-thickness bowel explants (1, 138) and endoscopic mucosal biopsy samples (139) of children with HSCR and others of various ages. These human cells can colonize embryonic bowel (127, 139), differentiate into some types of neuron and glia in appropriate positions, and restore some contractile function (127) in grafting studies. Many criteria have been used to enrich cells isolated from bowel for ENS stem cells, including RET expression (145), selection using reporters recapitulating the expression patterns of ENS genes (44, 45, 91), selection for proliferative capacity in culture (20), and coexpression of p75NTR and the HNK-1 carbohydrate epitope (207). Another well-defined population of stem cells present in embryonic and postnatal bowel of rats coexpress α_4_-integrin and high levels of p75NTR and is multipotent and self-renewing (14, 115) in culture. Many challenges lie between our current capability to expand a population of progenitors and the prospect of colonizing neonatal aganglionic bowel. Engraftment and migration of grafted cells through nonembryonic bowel have been quite limited (139, 196), and it is unclear what functional capabilities these cells could have once engrafted, although they do extend neuronal processes. One aspect that has received less attention is the use of ENS-derived stem cells for transplant into the CNS. These ENS-derived stem cells may be an ideal therapeutic source, since they are already capable of differentiating into cells expressing neurotransmitters lost in adult nervous system diseases. Furthermore, human ENS stem cells derived from a patient's own mucosal biopsies are proliferative, neurogenic, and nonimmunogenic without the need for genetic modification. They may be the most easily accessible neuronal stem cell in the body, and their use in CNS and ENS transplantation is worth investigating.

Although much effort has been focused on isolation and growth of ENS-derived stem cells in culture, these cells may serve a homeostatic role in postnatal ENS development, possibly in response to injury and aging. Recent work has demonstrated the existence of an extraganglionic cell that responds to 5-HT_4_ receptor stimulation by proliferating, becoming immunoreactive for SOX10, Phox2B, and HuC/HuD, and very slowly migrating into ganglia (130). Two recent studies used lineage tracing to demonstrate that enteric glia in the adult rat and mouse ENS have significant neurogenic potential in culture but only form neurons in vivo under very restricted circumstances. Adult cells labeled by an inducible recombinase under control of SOX10 genomic sequences (*Sox10-CreERT2*) never became neurons in vivo, except after ENS injury by benzalkonium chloride (121). However, a simultaneous study by another group demonstrated that neurogenesis from cells labeled by a GFAP-controlled recombinase (GFAP-Cre) did not occur after the same type of ENS injury. Furthermore, they did not detect proliferative neurogenesis in adult mice and rats exposed to an array of chemical, physical, infectious, and dietary insults (105). Taken together, these studies suggest that some neurogenesis in the adult ENS can occur via proliferation of an extraganglionic cell after 5-HT_4_ receptor stimulation or possibly through nonproliferative differentiation of an as-yet-unidentified SOX10-positive, GFAP-negative (or GFAP-Cre transgene-non-expressing) cell after injury. Further work is needed to identify the source of cells for both of these fascinating processes. Since neuronal progenitors within the postnatal and adult CNS express glial markers (114), the population currently considered to be uniformly enteric glia may contain a distinct subpopulation with the capacity to generate neurons.

## Prevention of HSCR and Other Intestinal Motility Disorders

While progress is being made toward novel transplantation strategies that might help treat HSCR or other serious motility disorders, HSCR prevention strategies deserve more focused study. Given the myriad of molecules and pathways involved in ENS development, it is very likely that one or more can be affected by some aspect of the prenatal environment. Counseling for parents of a child with sporadic HSCR is limited to providing information about the sibling recurrence risk, which varies depending on the sex of the proband and the length of aganglionosis. *RET* sequencing in HSCR patients is also becoming more common, since 1–2% of children that present with HSCR actually have *RET* mutations that cause MEN2A. However, knowing the nature of the mutation does not influence the treatment of HSCR.

Since treatment for HSCR remains imperfect and even diagnosed and treated HSCR causes significant morbidity, identifying environmental factors that could modify disease penetrance or expressivity would be extremely valuable. The vast majority of sporadic HSCR (80%) occurs because ENCDCs fail to colonize the final 5–10% of the bowel. At this critical point, small effects on ENCDC migration efficiency, proliferation, or survival can mean the difference between a functional colon and an aganglionic bowel causing life-threatening disease. By identifying and eliminating environmental factors that impair ENS development, we may be able to prevent some cases of short-segment disease and reduce the morbidity of more extensive aganglionosis. Very few associations between environmental factors and ENS development have been found, but this has not been systematically investigated. Only a few small clinical studies address whether the prenatal environment affects HSCR risk. One study of children with trisomy 21, for example, found that consumption of more than three cups of coffee a day and possibly maternal fever were associated with increased HSCR occurrence (195). An earlier study performed before the identification of any HSCR susceptibility genes also proposed an association between HSCR and hyperthermia during gestation (128), although a subsequent study failed to find a correlation (122). More subtle disorders of intestinal motility may also be rooted in environmental disruption of ENS development. In a recent retrospective study (149), tricyclic antidepressant use during the first trimester of pregnancy and selective 5-HT reuptake inhibitor use during the second or third trimester of pregnancy were associated with increases in laxative use (a surrogate for constipation) during early childhood. This is especially interesting, because tricyclic antidepressants inhibit the function of many receptors and transporters, including the norepinephrine transporter, which, as discussed previously, is required for normal TC-lineage differentiation into serotonergic neurons. In turn, 5-HT reuptake inhibitors might interfere with the normal role of 5-HT in later neurogenesis.

Animal models and culture studies provide strong evidence that specific gene-environment interactions influence ENS development and/or HSCR risk. Treatment of cultured fetal mouse colon with the Rho kinase inhibitor Y-27632, for example, inhibited ENCDC migration significantly more in *Re*t^+/−^ explants than in controls (185). Furthermore, oxidative stress in the early NC, induced by injection of pregnant mice with H_2_O_2_, reduced the extent of ENCDC migration into distal bowel in *Tcof1*^+/−^ embryos but did not affect the extent of ENCDC bowel colonization in wild-type littermates (9). A dramatic and clinically relevant gene-environment interaction was also observed in a mouse model of vitamin A deficiency (65). Mice maintain significant stores of vitamin A in their livers in the form of retinol, so *Rbp4*^−/−^ mice, which cannot mobilize these stores and depend on dietary retinol, were used to assess the effects of vitamin A depletion during ENS development. *Rbp4*^−/−^ mice fed a vitamin A-deficient diet during NC development had striking ENCDC migration delays in the colon compared with *Rbp4*^−/−^ mice fed a diet containing vitamin A. Additionally, *Rbp4*^−/−^, *Ret*^+/−^ mice had a much more severe delay in ENCDC colonization of the bowel when deprived of dietary vitamin A and even manifested a significant developmental delay when fed a vitamin A-sufficient diet. Similarly, in humans carrying HSCR risk alleles, otherwise subclinical vitamin A deficiency could synergize with genetic defects to worsen the severity of HSCR or increase the likelihood that HSCR will occur. Genetic models of HSCR susceptibility that more closely approximate sporadic HSCR and a careful examination of the signals involved in ENS development will be critical for identification and characterization of other environmental insults that impair ENS development and testing prevention strategies.

Finally, large-scale human epidemiological studies are appropriate and will be needed to validate and identify nongenetic factors that increase HSCR risk. Given the strength of the experimental data demonstrating that nongenetic factors can alter HSCR risk, the known effect of many medicines on proteins needed for ENS development, and our ability to couple genetic and epidemiological data, this is the ideal time to launch a systematic national or international case-control study of nongenetic HSCR risk factors. The implications of this work will have immediate benefit to families, since many children with HSCR are now becoming parents, and families who have one affected child are at dramatically higher risk of having a second child with HSCR.

## Conclusion

Much of our understanding of ENS development has been informed by developmental and genetic studies of very severe ENS defects, in a generally successful effort to understand the etiology of HSCR and ENCDC colonization of the bowel. However, we have highlighted several areas where aspects of global ENS development (cell motility, colonization, cell death, and gene-environment interactions) and processes with more restricted effects (neuronal fate decisions, axon pathfinding, and postnatal ENS stem cells) remain unexplained. To address these gaps in our understanding, it will be necessary to find new and more precise ways to perturb ENS development in experimental systems and expand the study of subtle and difficult-to-identify ENS phenotypes. Understanding normal ENS development and its modes of failure will translate into better outcomes for those affected by developmental defects of the ENS, whether these improvements come in the form of more informative genetic counseling, prevention strategies to mitigate the penetrance and expressivity of mutations, or stem cell therapy.

## GRANTS

This work was supported by National Institute of Diabetes and Digestive and Kidney Diseases Grant R01 DK-087715, Burroughs Wellcome Fund Clinical Scientist Award in Translational Research 1008525, Children's Discovery Institute of Washington University and St. Louis Children's Hospital Grants CH-II-1008-23, CH-II-2010-390, and MD-II-2013-269, and Washington University Research Strategic Alliance Grant 120178.

## DISCLOSURES

No conflicts of interest, financial or otherwise, are declared by the authors.

## AUTHOR CONTRIBUTIONS

J.I.L. prepared the figures; J.I.L. drafted the manuscript; J.I.L. and R.O.H. edited and revised the manuscript; J.I.L. and R.O.H. approved the final version of the manuscript.
